# ﻿Multigene phylogeny and morphology reveal *Ophiocordycepshydrangea* sp. nov. and *Ophiocordycepsbidoupensis* sp. nov. (Ophiocordycipitaceae)

**DOI:** 10.3897/mycokeys.92.86160

**Published:** 2022-08-30

**Authors:** Weiqiu Zou, Dexiang Tang, Zhihong Xu, Ou Huang, Yuanbing Wang, Ngoc-Lan Tran, Hong Yu

**Affiliations:** 1 Yunnan Herbal Laboratory, College of Ecology and Environmental Sciences, Yunnan University, Kunming 650504, Yunnan, China; 2 School of Life Science, Yunnan University, Kunming 650504, Yunnan, China; 3 Institute of Regional Research and Development, Ministry of Science and Technology, Hanoi, Vietnam

**Keywords:** 2 new taxa, entomopathogenic fungi, morphology, phylogenetic analyses

## Abstract

*Ophiocordyceps* species have a wide range of insect hosts, from solitary beetle larva to social insects. However, among the species of *Ophiocordyceps*, only a few attack cicada nymphs. These species are mainly clustered in the *Ophiocordycepssobolifera* clade in *Ophiocordyceps*. A new entomopathogenic fungus parasitic on cicada nymphs, and another fungus parasitic on the larva of Coleoptera, are described in this study. The two new species viz. *Ophiocordycepshydrangea* and *Ophiocordycepsbidoupensis* were introduced based on morphology and multigene phylogenetic evidence. The phylogenetic framework of *Ophiocordyceps* was reconstructed using a multigene (nr*SSU*, nr LSU, *tef-1*α, *rpb1*, and *rpb2*) dataset. The phylogenetic analyses results showed that *O.hydrangea* and *O.bidoupensis* were statistically well-supported in the *O.sobolifera* clade, forming two separate subclades from other species of *Ophiocordyceps*. The distinctiveness of these two new species was strongly supported by both molecular phylogeny and morphology.

## ﻿Introduction

*Ophiocordyceps* G.H. Sung, J.M. Sung, Hywel-Jones & Spatafora is the largest genus in the Ophiocordycipitaceae, comprising approximately 290 species. It was originally established by Petch, with *Ophiocordycepsblattae* Petch as the type species (Petch 1931). According to the arrangement of the perithecia, the size of asci, ascospores, and secondary ascospores, *Ophiocordyceps* was transferred to Cordyceps sensu lato by Kobayasi, as a subgenus of Cordyceps s.l. (Kobayasi 1941, 1982). Sung et al. (2007) used five to seven loci combined molecular datasets to revise the *Cordyceps* and the Clavicipitaceae. The species of *Cordyceps* and Clavicipitaceae were divided into three families (Cordycipitaceae, Ophiocordycipitaceae, Clavicipitaceae sense stricto) and four genera (*Cordyceps* sense stricto, *Ophiocordyceps*, *Elaphocordyceps*, and *Metacordyceps*). The research results of Sung et al. (2007) are currently the most widely accepted phylogenetic classification of *Cordyceps* s.l. In 2015, *Ophiocordyceps* was divided into *O.ravenelii* clade, *O.unilateralis* clade, *O.sobolifera* clade, and *O.sphecocephala* clade by Sanjuan et al. With the continuous revision of *Ophiocordyceps*, it has now been divided into four clades, including the *Hirsutella* clade, *O.sobolifera* clade, *O.sphecocephala* clade, and *O.ravenelii* clade (Mains 1958; Sung et al. 2007; Quandt et al. 2014; Sanjuan et al 2015; Simmons et al. 2015; Wang et al. 2018). Many phylogenetic classifications for entomopathogenic fungi have been revised in recent studies (Wang et al. 2018; Fan et al. 2021; Wang et al. 2021a, 2021b).

There are fewer species in the *O.sobolifera* clade than in the *Hirsutella* clade and the *O.sphecocephala* clade. The *O.sobolifera* clade is statistically well-supported in most studies and 11 species have been described in the Index Fungorum (Kobayasi and Shimizu 1963; Hywel-Jones 1995b; Sung et al. 2007, 2011; Luangsa-ard et al. 2008; Hyde et al. 2017; Crous et al. 2018, 2019; Lao et al. 2021; Wang et al. 2021a). Asexual morphs of *Ophiocordyceps* were reported as *Hirsutella* Pat., *Paraisaria* Samson & B.L. Brady, *Sorosporella* Sorokin, *Hymenostilbe* Petch and *Syngliocladium* Petch, etc. (Sung et al. 2007; Quandt et al. 2014). In most species of *Ophiocordyceps*, their dominant asexual morphs were *Hirsutella*, the conidiogenous cells basally swollen that taper to a narrow neck, producing a mucilaginous cluster of one or several conidia (Simmons et al. 2015; Wang et al. 2018).

*Ophiocordyceps* species have a wide range of insect hosts, from solitary beetle larvae to social insects. More than 10 insect orders were attacked, including Hemiptera, Coleoptera, Lepidoptera, Blattaria, Dermaptera, Diptera, Hymenoptera, Isoptera, Megaloptera, and Mantodea (Araújo et al. 2015; Araújo and Hughes 2016, 2019). Entomopathogenic fungi whose hosts are cicada nymphs have attractive stromata. The most typical representative of this group was *Cordycepscicadae* (Miquel) Massee (Massee 1895) in Cordycipitaceae, with the stroma like a flower (Sung et al. 2007). However, for species of *Ophiocordyceps*, with cicada nymph hosts including *O.khonkaenensis* Tasanathai, Thanakitpipattana & Luangsa-ard (Crous et al. 2019), *O.sobolifera* (Hill ex Watson) G.H. Sung, J.M. Sung, Hywel-Jones & Spatafora (Kobayasi and Shimizu 1963; Sung et al. 2007), and *O.longissima* (Kobayasi) G.H. Sung, J.M. Sung, Hywel-Jones & Spatafora (Kobayasi and Shimizu 1963; Sung et al. 2007, 2011) in *O.sobolifera* clade, their stromata were typically bright-colored and cylindrical. The hosts of the entomopathogenic fungi within the *O.sobolifera* clade were divided into two categories. One group with Hemiptera hosts was represented by *O.sobolifera.* These fungi had a hard texture stroma, which was cylindrical, and deep-colored, and had swollen fertile parts (Kobayasi and Shimizu 1963; Sung et al. 2011; Crous et al. 2019). Another group had Coleoptera hosts that were characterized by hard texture stromata, being cylindrical, bright-colored, and with a sterile apices cone at the top of the stroma (Hywel-Jones 1995b; Luangsa-ard et al. 2008; Crous et al. 2018; Lao et al. 2021; Wang et al. 2021a).

*Cordyceps* s.l. is globally distributed with the highest species diversity recorded in subtropical and tropical regions (Nguyen and Vo 2005; Ban et al. 2015; Doan et al. 2017; Luangsa-ard et al. 2018), especially in East and Southeast Asia (Sung et al. 2007; Fan et al. 2021; Wang et al. 2021a). To date, more than 800 species of *Cordyceps* and *Ophiocordyceps* have been named worldwide, and there are at least 200 species in China (Index Fungorum 2022). Yunnan Province, located in southwest China, has unique geographical and ecological features. Many species of *Ophiocordyceps* were reported from Yunnan, including *O.alboperitheciata* H. Yu, Q. Fan & Y.B. Wang (Fan et al. 2021), *O.furcatosubulata* H. Yu, Y. Wang & Y.B. Wang (Wang et al. 2021a), *O.highlandensis* Zhu L. Yang & J. Qin (Yang et al. 2015), *O.lanpingensis* H. Yu & Z.H. Chen (Chen et al. 2013), *O.laojunshanensis* J.Y. Chen, Y.Q. Cao & D.R. Yang (Chen et al. 2011), *O.liangshanensis* (M. Zang, D.Q. Liu & R.Y. Hu) H. Yu, Y. Wang, Y.D. Dai, Zhu L. Yang & Y.B. Wang (Wang et al. 2021b), and *O.pingbianensis* H. Yu, S.Q. Chen & Y.B. Wang (Chen et al. 2021). The unique geographical conditions of Yunnan have resulted in high *Cordyceps* s.l. species diversity. There is also a high species diversity of *Cordyceps* s.l. in Southeast Asia, where more than 500 species of entomopathogenic fungi have been reported. Approximately 400 species of entomopathogenic fungi are distributed in Thailand (Sung et al. 2007; Luangsa-ard et al. 2011, 2018; Ban et al. 2015; Tasanathai et al. 2019; Xiao et al. 2019). Vietnam is second to Thailand, in the number of entomopathogenic fungi species, with more than 100 species having been reported such as *Moelleriellapumatensis* T.T. Nguyen & N.L. Tran (Mongkolsamrit et al. 2011), *O.furcatosubulata* H. Yu, Y. Wang & Y.B. Wang (Wang et al. 2021a), and *O.puluongensis* H. Yu, Z.H. Xu, N.L. Tran & Y.B. Wang (Xu et al. 2022). These findings suggested that Vietnam should be abundant in species diversity of *Cordyceps* s.l. (Mongkolsamrit et al. 2011; Doan et al. 2017; Luyen et al. 2017).

Several studies have evaluated the taxonomy and biology of entomopathogenic fungi, especially species found in China and Southeast Asia. In this study, one unknown species of *Ophiocordyeps* attacking a cicada nymph was collected from Yunnan Province, Jinghong City, Nabanhe National Nature Reserve, in China. Another unknown species of *Ophiocordyeps* attacking larvae of Elateridae was collected from Lintong Province, Bidoup Nuiba National Park, in Vietnam. The phylogeny and morphology of these two fungi were determined, and their systematic position was established in Ophiocordycipitaceae. The phylogenetic analyses results showed that the two new species belonged to *Ophiocordyceps*, and were named *Ophiocordycepshydrangea* and *Ophiocordycepsbidoupensis* based on well-supported morphology and molecular data.

## ﻿Materials and methods

### ﻿Sample collection and isolation

The specimens were collected from China and Vietnam, and the collection site information was noted, including altitude, longitude, latitude, and habitat type. Samples were placed in sterilized tubes or plastic bags and boxes, returned to the laboratory, and stored at 4 °C. The specimens were photographed using a Canon 750 D camera (Canon Inc., Tokyo, Japan). The size was measured, and characteristics were recorded including length of the stroma, single or multiple, length and width of stipe clavate and fertile parts, shape, texture, and color. To obtain axenic cultures, the segments were removed from insect bodies, and these segments were placed onto Potato Dextrose Agar (PDA) consisting of peptone and yeast powder (potato 100 g/500 mL, dextrose 10 g/500 mL, agar 10 g/500 mL, yeast powder 5 g/500 mL, peptone 2.5 g/500 mL) plates. The plates were placed in a culture room at 25 °C until isolated into pure cultures. The cultures were saved on a PDA slant (to grow slowly), and stored at 4 °C. All specimens were deposited in the Yunnan Herbal Herbarium (YHH) of Yunnan University. The extypes of the two species were deposited in the Yunnan Fungal Culture Collection (YFCC) of Yunnan University.

### ﻿Morphological observations

To describe the sexual morphs of the two species, frozen sections or hand sections of the fruiting structures of the stroma were immersed in water and then dyed with lactophenol cotton blue solution for morphological observation and photomicrography (Wang et al. 2021a). For observations on asexual morphs, new colonies were established from old cultures and placed on new PDA plates. The plates were cultured in an incubator for 6 or 12 weeks at 25 °C, and then asexual morphs were observed and recorded (shape, texture, and color of the colonies). Microscope slide cultures were made using the methods of Wang et al. (2020). The morphological observations and measurements were made using Olympus CX40 and BX53 microscopes.

### ﻿DNA extraction, PCR, and sequencing

Five-centimeter segments from the stroma of fresh specimens and the cultures were used for DNA extraction to ensure the cultures and specimens were the same. Total DNA was extracted using cetyltrimethyl ammonium bromide (CTAB) according to the procedure described by Liu et al. (2001). The DNA was used for PCR amplification. The primer pair, NS4 (5'-CTTCCGTCAATTCCTTTAAG-3') and NS1 (5'-GTAGTCATATGCTTGTCTC-3') was used to amplify nr*SSU* (the nuclear ribosomal small subunit) (White et al. 1990). The primer pair, LR5 (5'-ATCCTGAGGGAAACTTC-3') and LR0R (5'-GTACCCGCTGAACTTAAGC-3') was used to amplify nr LSU (the nuclear ribosomal large subunit) (Vilgalys and Hester 1990; Rehner and Samuels 1994). The primer pair, 983F (5'-GCYCCYGGHCAYCGTGAYTTYAT-3') and 2218R (5'-ATGACACCRACRGCRACRGTYTG-3') was used to amplify *tef-1*α (the translation elongation factor 1α) (Rehner and Buckley 2005). The primer pair, CRPB1A (5'-CAYCCWGGYTTYATCAAGAA-3') and RPB1C (5'-CCNGCDATNTCRTTRTCCATRTA-3') were used to amplify *rpb1* (the largest subunit of RNA polymerase II) (Castlebury et al. 2004; Bischoff et al. 2006). The primer pair, fRPB2-5F (5'-GAYGAYMGWGATCAYTTYGG-3') and fRPB2-7cR (5'-CCCATRGCTTGYTTRCCCAT-3') was used to amplify *rpb2* (the second largest subunit of RNA polymerase II) (Liu et al. 1999). The polymerase chain reaction (PCR) for amplification of the five genes and their sequencing were described by Wang et al. (2015).

### ﻿Phylogenetic analyses

Sequences of the five genes (nr*SSU*, nr LSU, *tef-1*α, *rpb1*, and *rpb2*) were downloaded from GenBank, and combined with the newly generated sequences in this study. The taxa information of the species and GenBank accession numbers of the five genes are listed in Table 1. Sequences of the five genes were aligned using the Clustal X (v.2.0) and MEGA6 (v.6.0) (Larkin et al. 2007; Tamura et al. 2013). Ambiguously aligned sites were eliminated, and the gaps were treated as missing data. The aligned sequences of the five genes (nr*SSU*, nr LSU, *tef-1*α, *rpb1*, and *rpb2*) were concatenated into a single combined dataset using MEGA6 (v.6.0.). Conflicts between the five genes were tested using PAUP* (v.4.0b10) (Swofford 2002). The results of the phylogenetic signals in the five genes were not in conflict. The concatenated dataset containing all five genes consisted of 11 data partitions, including one each for nr*SSU* and nr LSU, and three for each of the three codon positions of *tef-1*α, *rpb1*, and *rpb2*. Phylogenetic analyses based on the five genes were made using BI and ML methods (Ronquist and Huelsenbeck 2003; Stamatakis et al. 2008). We used the optimal model GTR+I with 1,000 rapid bootstrap replicates on the five genes for ML analyses (Stamatakis 2006). We conducted BI analyses using a GTR+G+I model determined by jModelTest (v.2.1.4), conducted on MrBayes (v.3.1.2) for 5 million generations (Darriba et al. 2012). The phylogenetic tree constructed was viewed and edited using FigTree (v.1.4.2) and Adobe Illustrator CS6.

**Table 1.** Specimen information and GenBank accession numbers of the sequences used in this study.

Species	Host	Isolate no./ specimen no.	GenBank accession no.				
			*nrS*SU	nr LSU	*tef-1*α	*rpb1*	*rpb2*
*Hirsutellacitriformis*	Cixiidae (Hemiptera)	ARSEF 1446	KM652065	KM652106	KM651990	KM652031	–
*Hirsutellafusiformis*	*Brachyderesincanus* (Curculionidae, Coleoptera)	ARSEF 5474	KM652067	KM652110	KM651993	KM652033	–
*Hirsutellagigantea*	Pamphiliidae (Hymenoptera)	ARSEF 30	–	JX566977	JX566980	KM652034	–
*Hirsutellaguyana*	*Empoascakraemeri* (Cicadellidae, Hemiptera)	ARSEF 878	KM652068	KM652111	KM651994	KM652035	–
*Hirsutellaillustris*	*Eriosomalanigerum* (Aphididae, Hemiptera)	ARSEF 5539	KM652069	KM652112	KM651996	KM652037	–
*Hirsutellakirchneri*	*Abacarushystrix* (Eriophyidae, Acari)	ARSEF 5551	KM652070	KM652113	KM651997	–	–
*Hirsutellalecaniicola*	*Parthenolecaniumcorni* (Coccidae, Hemiptera)	ARSEF 8888	KM652071	KM652114	KM651998	KM652038	–
*Hirsutellaliboensis*	Larva of Cossidae (Lepidoptera)	ARSEF 9603	KM652072	KM652115	KY415588	KY945367	–
*Hirsutellanecatrix*	Acari	ARSEF 5549	KM652073	KM652116	KM651999	KM652039	–
*Hirsutellanodulosa*	*Dioryctriazimmermani* (Pyralidae, Lepidoptera)	ARSEF 5473	KM652074	KM652117	KM652000	KM652040	–
*Hirsutellaradiata*	Diptera	ARSEF 1369	KM652076	KM652119	KM652002	KM652042	–
*Hirsutellarhossiliensis*	*Mesocriconemaxenoplax* (Criconematidae, Tylenchida)	ARSEF 3747	KM652080	KM652123	KM652006	KM652045	–
*Hirsutellastrigosa*	*Nephotettixvirescens* (Cicadellidae, Hemiptera)	ARSEF 2197	KM652085	KM652129	KM652012	KM652050	–
*Hirsutellasubulata*	Microlepidoptae (Lepidoptera)	ARSEF 2227	KM652086	KM652130	KM652013	KM652051	–
Hirsutellathompsoniivar.synnematosa	*Aceriasheldoni* (Eriophyidae, Acari)	ARSEF 2459	KM652099	KM652147	KM652027	KM652061	–
Hirsutellathompsoniivar.thompsonii	*Phyllocoptrutaoleivora* (Eriophyidae, Acari)	ARSEF 137	KM652087	KM652131	KM652014	KM652052	–
Hirsutellathompsoniivar.vinacea	*Acalitusvaccinii* (Eriophyidae, Acari)	ARSEF 254	KM652101	KM652149	KM652028	KM652062	–
*Ophiocordycepsacicularis*	Larva of Coleoptera	OSC 110987	EF468950	EF468805	EF468744	EF468852	–
*Ophiocordycepsacicularis*	Larva of Coleoptera	OSC 110988	EF468951	EF468804	EF468745	EF468853	–
*Ophiocordycepsagriotidis*	Larva of Coleoptera	ARSEF 5692	DQ522540	DQ518754	DQ522322	DQ522368	DQ522418
*Ophiocordycepsannulata*	Larva of Coleoptera	CEM 303	KJ878915	KJ878881	KJ878962	KJ878995	–
*Ophiocordycepsaphodii*	Larva of Scarabaeidae (Coleoptera)	ARSEF 5498	DQ522541	DQ518755	DQ522323	–	DQ522419
*Ophiocordycepsappendiculata*	Larva of Coleoptera	NBRC 106960	JN941728	JN941413	AB968577	JN992462	AB968539
*Ophiocordycepsarborescens*	Larva of *Puerarialobata* (Lepidoptera)	NBRC 105891	AB968386	AB968414	AB968572	–	AB968534
***Ophiocordycepsbidoupensis***	**Larva of Elateridae (Coleoptera)**	**YFCC 8793**	**OM304638**	–	**OK556894**	**OK556898**	**OK556900**
***Ophiocordycepsbidoupensis***	**Larva of Elateridae (Coleoptera)**	**YHH 20036**	**OK571396**	–	**OK556893**	**OK556897**	**OK556899**
*Ophiocordycepsbrunneanigra*	Cicadellidae (Hemiptera)	TBRC 8093	–	MF614654	MF614638	MF614668	MF614681
*Ophiocordycepsbrunneaperitheciata*	Larva of Lepidoptera	TBRC 8100	–	MF614658	MF614643	–	MF614685
*Ophiocordycepsbrunneipunctata*	Larva of Elateridae (Coleoptera)	OSC 128576	DQ522542	DQ518756	DQ522324	DQ522369	DQ522420
*Ophiocordycepscitrina*	Hemiptera	TNS F18537	–	KJ878903	KJ878983	–	KJ878954
*Ophiocordycepscochlidiicola*	Cochlidiidae pupa (Lepidoptera)	HMAS 199612	KJ878917	KJ878884	KJ878965	KJ878998	–
*Ophiocordycepscossidarum*	Larva of Cossidae (Lepidoptera)	MFLU 17-0752	MF398186	MF398187	MF928403	MF928404	–
*Ophiocordycepscrinalis*	Larva of Lepidoptera	GDGM 17327	KF226253	KF226254	KF226256	KF226255	–
*Ophiocordycepsevansii*	*Pachycondylaharpax* adult ant (Hymenoptera)	HUA 186159	KC610796	KC610770	KC610736	KP212916	–
*Ophiocordycepsformicarum*	Formicidae (Hymenoptera)	TNS F18565	KJ878921	KJ878888	KJ878968	KJ879002	KJ878946
*Ophiocordycepsforquignonii*	Adult fly (Diptera)	OSC 151902	KJ878912	KJ878876	–	KJ878991	KJ878945
*Ophiocordycepsfurcatosubulata*	Larva of Elateridae (Coleoptera)	YFCC 904	MT774216	MT774223	MT774244	MT774230	MT774237
*Ophiocordycepsfurcatosubulata*	Larva of Elateridae (Coleoptera)	YHH 17005	MT774217	MT774224	MT774245	MT774231	MT774238
*Ophiocordycepsgeometridicola*	Larva of Geometridae (Lepidoptera)	TBRC 8095	–	MF614648	MF614632	MF614663	MF614679
*Ophiocordycepshouaynhangensis*	Larva of Coleoptera	TBRC 8428	–	MH092902	MH092894	–	–
***Ophiocordycepshydrangea***	**Nymph of cicada (Hemiptera)**	**YFCC 8832**	**OM304636**	**OM304640**	**OM831277**	**OM831280**	**OM831283**
***Ophiocordycepshydrangea***	**Nymph of cicada (Hemiptera)**	**YFCC 8833**	**OM304637**	**OM304641**	**OM831278**	**OM831281**	**OM831284**
***Ophiocordycepshydrangea***	**Nymph of cicada (Hemiptera)**	**YFCC 8834**	**OM304635**	**OM304639**	**OM831276**	**OM831279**	**OM831282**
*Ophiocordycepskarstii*	*Hepialusjianchuanensis* (Lepidoptera)	MFLU:15-3884	KU854952	–	KU854945	KU854943	–
*Ophiocordycepskimflemingiae*	*Camponotus castaneus/americanus* (Hymenoptera)	SC09B	KX713631	KX713620	KX713698	KX713724	–
*Ophiocordycepskniphofioides*	*Cephalotesatratus* adult ant (Hymenoptera)	HUA 186148	KC610790	KF658679	KC610739	KF658667	KC610717
*Ophiocordycepskonnoana*	Larva of Coleoptera	EFCC 7315	EF468959	–	EF468753	EF468861	EF468916
*Ophiocordycepslangbianensis*	Larva of Coleoptera	DL0017	MT928355	MT928306	–	–	–
*Ophiocordycepslanpingensis*	Larva of Hepialidae (Lepidoptera)	YHOS0705	KC417458	KC417460	KC417462	KC417464	KC456333
*Ophiocordycepslongissima*	Cicada nymph (Cicadidae, Hemiptera)	NBRC 106965	AB968392	AB968420	AB968584	–	AB968546
*Ophiocordycepslongissima*	Hemiptera; cicada (nymph)	EFCC 6814	–	EF468817	EF468757	EF468865	–
*Ophiocordycepsmacroacicularis*	Larva of Cossidae (Lepidoptera)	NBRC 100685	AB968388	AB968416	AB968574	–	AB968536
*Ophiocordycepsmultiperitheciata*	Lepidoptera larva	BCC 69008	–	MF614657	MF614641	–	MF614682
*Ophiocordycepsmyrmicarum*	Hymenoptera (Formicidae)	HIRS 45	KJ680150	JX566965	JX566973	KJ680151	–
*Ophiocordycepsnigrella*	Larva of Lepidoptera	EFCC 9247	EF468963	EF468818	EF468758	EF468866	EF468920
*Ophiocordycepspruinosa*	Hemiptera	NHJ 12994	EU369106	EU369041	EU369024	EU369063	EU369084
*Ophiocordycepspseudoacicularis*	Larva of Lepidoptera	TBRC 8102	–	MF614646	MF614630	MF614661	MF614677
*Ophiocordycepspulvinata*	*Camponotus* adult ant (Hymenoptera)	TNS-F 30044	GU904208	AB721305	GU904209	GU904210	–
*Ophiocordycepsramosissimum*	*Phassusnodus* larva (Lepidoptera)	GZUHHN8	KJ028012	–	KJ028014	KJ028017	–
*Ophiocordycepsravenelii*	Beetle larva (Coleoptera)	OSC 110995	DQ522550	DQ518764	DQ522334	DQ522379	DQ522430
*Ophiocordycepsrobertsii*	Larva of Hepialidae (Lepidoptera)	KEW 27083	–	EF468826	EF468766	–	–
*Ophiocordycepsrubiginosiperitheciata*	Larva of Coleoptera	NBRC 106966	JN941704	JN941437	AB968582	JN992438	AB968544
*Ophiocordycepssatoi*	*Polyrhachislamellidens* (Hymenoptera)	J19	KX713650	KX713601	KX713684	KX713710	–
*Ophiocordycepssinensis*	Larva of Hepialidae (Lepidoptera)	EFCC 7287	EF468971	EF468827	EF468767	EF468874	EF468924
*Ophiocordycepssinensis*	Larva of Hepialidae (Lepidoptera)	YHH 1805	MK984568	MK984580	MK984572	MK984587	MK984576
*Ophiocordycepssobolifera*	Cicada nymph (Cicadidae, Hemiptera)	TNS F18521	KJ878933	KJ878898	KJ878979	KJ879013	–
*Ophiocordycepssobolifera*	Hemiptera (cicada nymph)	NBRC 106967	AB968395	AB968422	AB968590	–	–
*Ophiocordycepsspataforae*	Hemiptera adult	NHJ 12525	EF469125	EF469078	EF469063	EF469092	EF469111
*Ophiocordycepssphecocephala*	Hymenoptera adult wasp	NBRC 101753	JN941695	JN941446	AB968592	JN992429	AB968553
*Ophiocordycepsstylophora*	Larva of Elateridae (Coleoptera)	OSC 110999	EF468982	EF468837	EF468777	EF468882	EF468931
*Ophiocordycepsthanathonensis*	Hymenotera adult ant	MFLU 16-2910	MF882926	MF850377	MF872614	MF872616	–
*Ophiocordycepstiputinii*	Larva of Megaloptera	QCNE 186287	KC610792	KC610773	KC610745	KF658671	–
*Ophiocordycepstricentri*	Adult of Cercopoidea (Hemiptera)	NBRC 106968	AB968393	AB968423	AB968593	–	AB968554
*Ophiocordycepsunilateralis* s. str.	Camponotus sericeiventris (Hymenoptera)	VIC 44303	KX713628	KX713626	KX713675	KX713730	–
*Ophiocordycepsunituberculata*	Larva of Lepidoptera	YFCC HU1301	KY923214	KY923212	KY923216	KY923218	KY923220
*Ophiocordycepsxuefengensis*	Larva of *Phassusnodus* (Lepidoptera)	GZUH2012HN14	KC631789	–	KC631793	KC631798	–
*Ophiocordycepsyakusimensis*	Cicada nymph (Cicadidae, Hemiptera)	HMAS 199604	KJ878938	KJ878902	–	KJ879018	KJ878953
*Paraisariaamazonica*	Adult of Acrididae (Orthoptera)	HUA 186143	KJ917562	KJ917571	KM411989	KP212902	KM411982
*Paraisariacoenomyiae*	*Coenomyia* sp. (Coenomyiidae, Diptera)	NBRC 106964	AB968385	AB968413	AB968571	–	AB968533
*Paraisariagracilis*	Larva of Lepidoptera	EFCC 8572	EF468956	EF468811	EF468751	EF468859	EF468912
*Paraisariaheteropoda*	Cicada nymph (Hemiptera)	NBRC 100644	JN941718	JN941423	AB968596	JN992452	AB968557
*Tolypocladiuminflatum*	Coleoptera (larva)	OSC 71235	EF469124	EF469077	EF469061	EF469090	EF469108
*Tolypocladiumophioglossoides*	Fungi (*Elaphomyces* sp.)	CBS 100239	KJ878910	KJ878874	KJ878958	KJ878990	KJ878944

## ﻿Results

### ﻿Phylogenetic analyses

**Figure 1.**
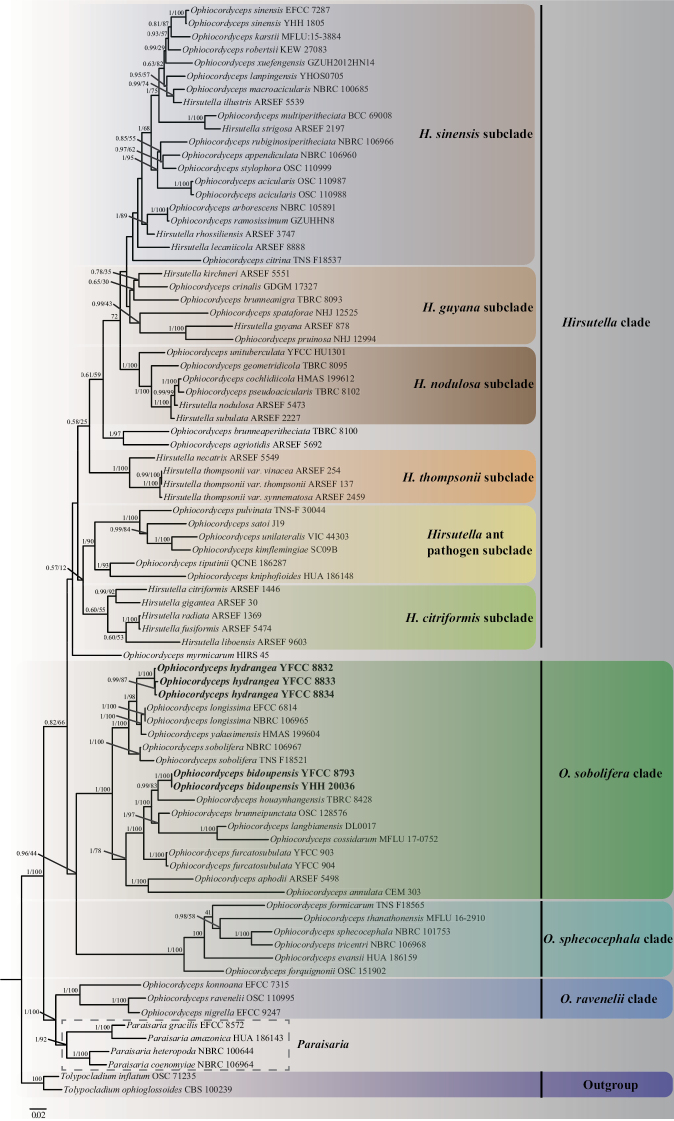
Phylogenetic relationships of *Ophiocordycepshydrangea* and related species from the five genes dataset (nr LSU, nr*SSU*, *tef-1*α, *rpb1*, and *rpb2*) based on ML and BI analyses. Statistical support values of BI posterior probabilities and ML bootstrap proportions (0.5/≥50%) are shown at the nodes.

A total of 83 samples were used for the phylogenetic analyses. Five gene sequences of the two new species collected were used to reconstruct the phylogenetic framework of *Ophiocordyceps*. Two taxa of *Tolypocladium* were designated as the outgroup, and these were, respectively, *Tolypocladiumophioglossoides* CBS 100239 and *Tolypocladiuminflatum* OSC 71235. The alignment lengths of the 83 samples were composed of 4,486 bp sequence data, 971 bp of nr*SSU*, 921 bp of nr LSU, 943 bp of *tef-1*α, 726 bp of *rpb1*, and 925 of *rpb2*. The phylogenetic tree showed that these were identical in overall topologies to previous studies. Four clades (*Hirsutella* clade, *O.sobolifera* clade, *O.sphecocephala* clade, and *O.ravenelii* clade) of *Ophiocordyceps* were well-supported by ML bootstrap proportions and BI posterior probabilities (Fig. 1). The two new species in the *O.sobolifera* clade, *O.hydrangea* and *O.bidoupensis*, formed two separate subclades. Three samples of *O.hydrangea* (BP = 98%, PP = 1) formed a separate subclade with *O.longissima* and *O.yakusimensis*, while *O.bidoupensis* (BP = 83%, PP = 0.99) formed a separate subclade with *O.houaynhangensis*.

## ﻿Taxonomy

### ﻿ Ophiocordyceps hydrangea

Taxon classificationFungiHypocrealesOphiocordycipitaceae

H. Yu, W.Q. Zou & D.X. Tang sp. nov.

43B87E78-7CCA-5FB0-BC2D-95185100F02E

MycoBank No: 843203

Fig. 2

#### Etymology.

Hydrangea, referred to the top of the stroma similar to hydrangea.

#### Holotype.

China, Yunnan Province, Jinghong City, Nabanhe National Nature Reserve, 22°8'21.32"N, 100°42'18.35"E, alt. 612 m, on cicada nymphs (Cicadidae, Hemiptera). The material was found in the soil of an evergreen broad-leaved forest, 18 August 2020, H. Yu (YHH 20081, holotype; YFCC 8834, ex-holotype culture).

**Figure 2.**
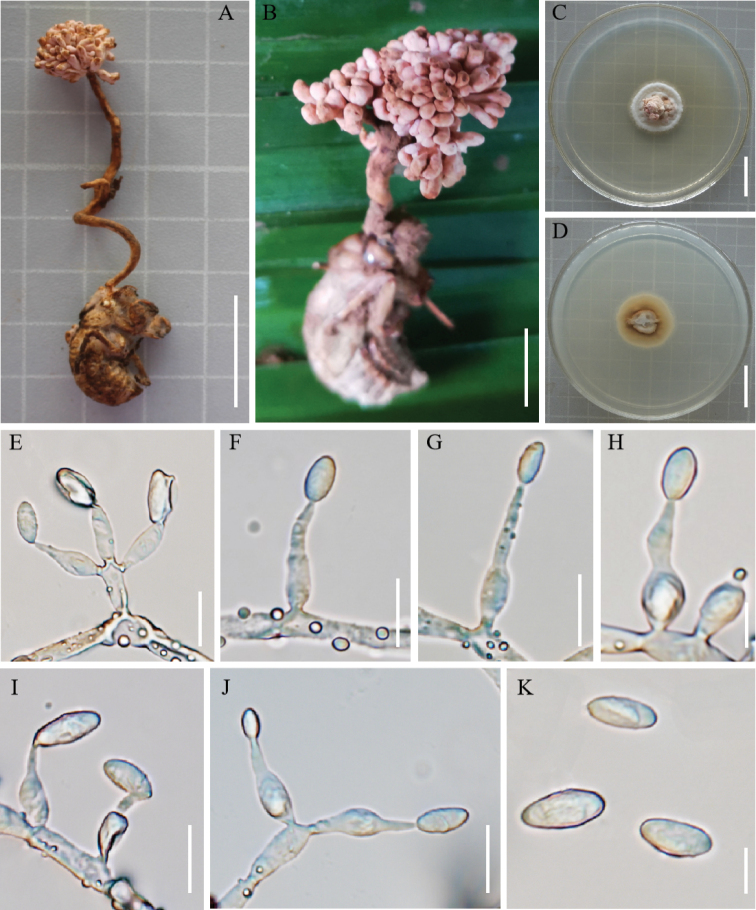
*Ophiocordycepshydrangea***A, B** fungus on a cicada nymph **C, D** colony on PDA medium **E** conidiophores, conidiogenous cells and conidia **F–J** conidiogenous cells and conidia **K** conidia. Scale bars: 1 cm (**A, B**); 2 cm (**C, D**); 10 µm (**E, F, G, I, J**); 5 µm (**H, K**).

#### Sexual morph.

The stroma was grown from the head of the host cicada nymph, solitary, the top of the stroma similar to hydrangea, pale pink, 1.6–6.4 cm long. Sexual morph was not observed.

#### Asexual morph.

The colony grew slowly on PDA medium. Cultured at 25 °C for about 12 weeks, the diameter of the colony was 25–28 mm, pale pink, the edge white, hard texture. The back of the colony was white to brown. Surface hyphae rough, hyaline, septate. Conidiophores were cylindrical. Conidiogenous cells were solitary or whorled, ampuliform, smooth-walled, forming on conidiophores or colonies, hyaline, with swollen base, and slender top, 10.6–17.6 µm long, 2.9–4.3 µm wide at the swollen base, and 1.1–2.2 µm wide at the slender top. Conidia hyaline, ovoid or long oval, solitary, 6.8–10.1 × 3.3–4.5 µm.

#### Host.

Cicada nymph (Cicadidae, Hemiptera).

#### Habitat.

In the soil of an evergreen broad-leaved forest.

#### Distribution.

China.

#### Other material examined.

China, Yunnan Province, Jinghong City, Nabanhe National Nature Reserve, 22°8'21.32"N, 100°42'18.35"E, alt. 612 m, on cicada nymphs (Cicadidae, Hemiptera) was found in the soil an evergreen broad-leaved forest, 18 August 2020, H. Yu (YFCC 8832, YFCC 8833).

#### Notes.

Phylogenetic analyses showed that *O.hydrangea* clustered with *O.sobolifera*, *O.longissima*, and *O.yakusimensis* of the *O.sobolifera* clade (Fig. 1). Their hosts were cicada nymphs compared to other species of the *O.sobolifera* clade (Table 2). *Ophiocordycepshydrangea* was well supported by BI and ML results, forming a separate subclade with *O.sobolifera*, *O.longissima*, and *O.yakusimensis*. The macro-morphology of *O.hydrangea* was clearly different from *O.sobolifera*, *O.longissima*, *O.khonkaenensis*, and *O.yakusimensis*. The stroma of *O.hydrangea* grew from the head of the host cicada nymph, solitary, and the top of the stroma was like a pale pink hydrangea.

**Table 2.** Morphological comparisons of two new species and related species.

Species	Host	stromata	Perithecia	Asci	Ascospores	Conidiogenous cells	Conidia	References
***O.bidoupensis***	**Larva of Elateridae (Coleoptera)**	**Solitary, solid, cylindrical, yellow, 11.8–22.5 cm long.**	**Immersed, pyriform to lanceolate, brown-yellow, 213.4–405.9 × 74.8–192.4 μm.**	**Hyaline, slender, 116.1–192.7 × 4.8–7.5 μm.**	**Hyaline, filiform, multi-septate.**	**Cone, hyaline, septate, smooth-walled, forming on hyphae, with a hypertrophic base, tapering abruptly into a thin neck, smooth-walled, 13.8–46.4 × 0.42–5.13 μm.**	**Oval or briolette, hyaline, smooth-walled, 2.24–3.61 × 1.49–2.70 μm.**	**This study**
*O.brunneipunctata*	Larva of Elateridae (Coleoptera)	Solitary, rarely up to 3, simple, 25–90 mm high.	Immersed, perithecioid, brown, ovate to pyriform, brown-walled, 270–335 × 110–160 μm.	Hyaline, cylindric, capitate, 8-spored, 280–295 × 6–7 μm.	Hyaline, filiform, multiseptate breaking into 64 part spores, 4–6 × 1–1.5 μm.	Monophialidic, rarely polyphialidic, hyaline, smooth, 5.5–7.5 × 2.5–3.0 μm at the base, up to 15 × 0.5 μm above.	Hyaline, aseptate, smooth, spherical 1.5–2.5 μm diam., enveloped by a mucous sheath.	Hywel-Jones 1995b; Luangsa-ard et al. 2008
*O.cossidarum*	Larva of Cossidae (Lepidoptera)	Solitary, simple, 40–70 mm high.	Immersed, red, ovate to phialide, red-walled, 355–454 × 136–171 μm.	Hyaline, cylindrical, 8-spored with a thickened apex, 174–221 × 5.7–7 μm.	Hyaline, fifiliform, multiseptate,131–153 × 1.8–2.2 μm, breaking into 32 part-spores.	–	–	Hyde et al. 2017
*O.furcatosubulata*	Larva of Elateridae (Coleoptera)	Single, solid, yellow to brown, 40–80 mm long, 1.5–2.2 mm wide.	Immersed, long ovoid or pyriform, 289.6–405.8 × 87.0–159.2 µm.	Hyaline, cylindrical, 138.8–202.5 × 4.3–6.0 μm.	Hyaline, filiform, multi-septate, finally breaking into secondary ascospores, 3.7–5.3 × 1.3–2.0 μm.	Polyphialidic, forming on conidiophores or side branches, hyaline, with a slender or subulate base, tapering gradually, smooth-walled or verruculose, 3.5–15.8 × 0.9–1.7 μm.	Solitary, aseptate, smooth-walled, broadly ellipsoid or ellipsoid, 1.5–2.5 × 1.2–1.9 μm.	Wang et al. 2021a
*O.houaynhangensis*	Larva of Coleoptera	Solitary, cylindrical, cream, up to 11 cm long and 1.5–2.5 mm in width.	Completely immersed, obclavate, 300–450 × 80–170 µm.	Cylindrical, 100–250 × 4–7.5 µm.	Hyaline, cylindrical, breaking into 32 small truncate part-spores, 4–7 × 1–2 µm.	Monophialidic, phialides flasked-shaped with long necks, up to 30 µm long and 2–4 µm in breadth; phialide necks up to 18 μm long and 0.5 µm in breadth.	Hyaline, smooth, spherical, 2–3 µm.	Crous et al. 2018
*O.langbianensis*	Larva of Coleoptera	Solitary, rarely branched, 40–100 mm long.	Immersed, ovate or pyriform, 260–400 × 100–190 µm.	Cylindrical, with thickened cap, 200–250 × 5.0–6.0 μm.	Fliform, multiseptate, articulated in long-chain afer discharging, sometimes breaking into 1-celled part spores, 5–7.5 × 1.3–2 µm.	Divergent.	Chains, elliptical.	Lao et al. 2021
*O.sobolifera*	Cicada nymph (Cicadidae, Hemiptera)	Commonly single, rarely fasciculated by twos or threes, arising from head among polster, clavate or cylindric 2–8 cm long, 2–6 mm thick, become hollow after maturity.	Rectangularly immersed, ampullaceous 500–600 × 220–260 μm, with somewhat long neck, ostiola somewhat prominent, walls hyaline 8–16 μm thick.	Cylindric, 400–470 × 5.6–6.3 μm.	Finally breaking into secondary ascospores, truncate at both ends, 6–12 × 1.0–1.3 μm.	–	Terminal or lateral, ellpsoid or fusiformed, hyaline, 6.5–10.5 × 2.5–4.0 μm.	Kobayasi and Shimizu 1963
*O.yakusimensis*	Cicada nymph (Cicadidae, Hemiptera)	Very long attaining 14 cm, arising from the apical part between eyes.	Wholly embeddèd, narrow ovoid or almost naviculate, 740–800 × 170–230 μm, without protruding ostiola, neck almost destitute, wall 21–23 μm thick, composed of very thin cells.	270–310 × 5 μm.	Finally breaking into secondary ascospores, long cylindrical, somewhat attenuated on both sides, terminally truncate, 10–15 × 1 μm.	–	–	Kobayasi and Shimizu 1963
*longissima*	Cicada nymph (Cicadidae, Homoptera)	5–20 cm long, some times much longer.	Ovoid to long ovoid, with a short neck, 440–590 × 130–300 µm.	190–350 × 5–6 µm.	–	–	–	Sung et al. 2011
*O.khonkaenensis*	Cicada nymph (Hemiptera)	Variable in number, solitary to three, 20–30 mm long and 2–3 mm in breath.	Immersed, flask shaped, 590–700 × 200–300 µm.	Cylindrical, 237.5–337.5 × 5–6 µm.	Filiform, 300–360 × 1–1.5 µm readily breaking into 32 part-spores, 7–13 × 1–1.5 µm.	Phialidic, hirsutella-like, 5.5–11 × 2–3 µm.	Hyaline, fusiform, smoothwalled, 3–5.5 × 1–3 µm.	Crous et al. 2019
***O.hydrangea***	**Cicada nymph (Cicadidae, Hemiptera)**	**Solitary, the top of the stroma similar to hydrangea, pale pink,1.6–6.4 cm long.**	–	–	–	**Solitary or whorled, ampuliform, smooth-walled, forming on conidiophores or colonies, hyaline, with swollen base, and slender top, 10.6–17.6 µm long, 2.9–4.3 µm wide at the swollen base, and 1.1–2.2 µm wide at the slender top.**	**Hyaline, ovoid or long oval, solitary, 6.8–10.1 × 3.3–4.5 µm**.	**This study**

### ﻿ Ophiocordyceps bidoupensis

Taxon classificationFungiHypocrealesOphiocordycipitaceae

H. Yu, W.Q. Zou & D.X. Tang sp. nov.

7F6718B3-1C1D-52CA-B72D-531CBD6AEA2A

MycoBank No: 843204

Fig. 3

#### Etymology.

Bidoupensis, referred to the type species collected from Bidoup Nuiba National Park.

#### Holotype.

Vietnam, Lintong Province, Bidoup Nuiba National Park, 12°8'9.30"N, 108°31'51.38"E, alt. 1678 m, on larva of Elateridae (Coleoptera) buried in soil, emerging from the leaf litter on the forest floor, 16 October 2017, H. Yu (YHH 20036, holotype; YFCC 8793, ex-holotype culture).

**Figure 3.**
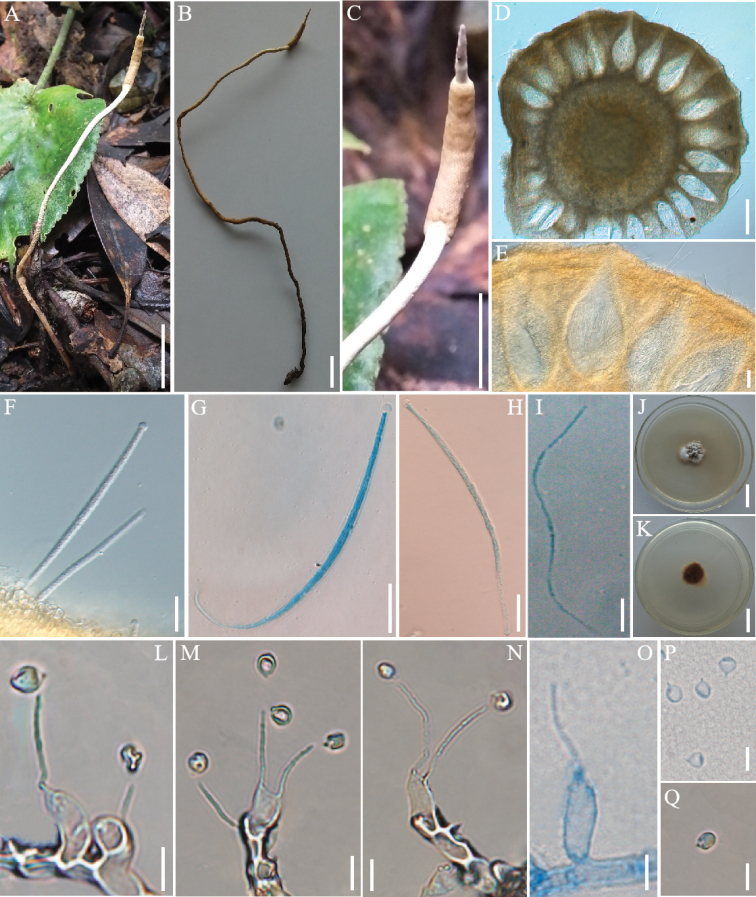
*Ophiocordycepsbidoupensis***A–C** fungus on an Elateridae larva **D, E** cross-section of the ascoma showing the perithecial arrangement **F–H** asci **I** ascospores **J, K** colony on PDA medium **L–N** conidiogenous cells and conidia **O** conidiogenous cells **P, Q** conidia. Scale bars: 1 cm (**A–C**); 200 µm (**D**); 20 µm (**E–H**); 10 µm (**I**); 2 cm (**J, K**); 5 µm (**L–Q**).

#### Sexual morph.

The stroma grew from the head of the host, solitary, solid, cylindrical, 11.8–22.5 cm long, yellow. Stipe clavate, yellow, curved, 10.7–21.2 cm long, 0.7–0.9 mm wide. Fertile parts cylindrical, yellow, slightly curved, 2.9–11.3 mm long, 0.9–1.6 mm wide. Sterile apices cone, yellow, 2.1–7.2 mm long, 0.2–0.7 mm wide. Perithecia immersed, pyriform to lanceolate, brown-yellow, 213.4–405.9 × 74.8–192.4 μm. Asci hyaline, slender, 116.1–192.7 × 4.8–7.5 μm. Asci cap prominent, capitate, 4.7–6.1 × 3.3–5.4 μm. Ascospores hyaline, filiform, multi-septate.

#### Asexual morph.

The colony grew slowly on PDA medium. Cultured at 25 °C for about 6 weeks, the diameter of the colony was 38–45 mm, white, aerial mycelium on the surface, slightly convex. The back of the colony was grayish-white, dark brown in the middle. Surface smooth of hyphae, hyaline, septate. Conidiogenous cells cone, hyaline, septate, smooth-walled, forming on hyphae, with a hypertrophic base, tapering abruptly to a thin neck, 13.80–46.4 × 0.42–5.13 μm. Conidia hyaline, oval or briolette, smooth-walled, 2.24–3.61 × 1.49–2.70 μm.

#### Host.

Larva of Elateridae (Coleoptera).

#### Habitat.

The hosts were buried in soil, and the stroma were found in the leaf litter on the forest floor.

#### Distribution.

Vietnam.

#### Notes.

Phylogenetic analyses showed that *O.bidoupensis* was clustered with *O.houaynhangensis*, *O.brunneipunctata*, *O.langbianensis*, *O.cossidarum*, and *O.furcatosubulata* of the *O.sobolifera* clade (Fig. 1). Their hosts were larvae of Elateridae compared to cicada nymph hosts of the other species of the *O.sobolifera* clade (Table 2). *Ophiocordyceosbidoupensis* was well-supported by bootstrap support and posterior probabilities, and formed a separate subclade with *O.houaynhangensis*, *O.brunneipunctata*, *O.langbianensis*, and *O.cossidarum*. The morphology of *O.bidoupensis* was clearly different in shape and size from other species of *O.sobolifera* clade (Table 2). The stroma of *O.bidoupensis* grew solitary from the head of the host; sterile apices of the stroma were different from the other species.

## ﻿Discussion

*Ophiocordyceps* is the largest genus in the Ophiocordycipitaceae, with a wide range of hosts and various species. At present, more than 290 species of *Ophiocordyceps* have been reported (Index Fungorum 2022). However, only 11 species are described in the *O.sobolifera* clade and their hosts are mainly Coleoptera larvae and cicada nymphs (Hemiptera) (Table 2). We describe the new species *O.hydrangea* attacking cicada nymphs and the new species *O.bidoupensis* attacking Coleoptera larvae. Most species have diverse macro-morphological or micro-morphological characteristics due to the same entomopathogenic fungi having a different host, or different species of entomopathogenic fungi having the same host (Sung et al. 2007, 2011; Araújo et al. 2015; Araújo and Hughes 2016; Shrestha et al. 2016; Luangsa-ard et al. 2018; Crous et al. 2019; Fan et al. 2021; Wang et al. 2021a). Hemiptera hosts are widely present among the species of *Ophiocordyceps*, including species of the *Hirsutella* clade, *O.sobolifera* clade, *O.sphecocephala* clade, and *O.ravenelii* clade.

The entomopathogenic fungi whose host is Hemiptera have diverse morphological characteristics. For example, *O.nutans* (Patouillard) G.H. Sung, J.M. Sung, Hywel-Jones & Spatafora (Sung et al. 2007), its hosts were stink bugs (Hemiptera), stromata solitary or multiple, fertile parts was red (Hywel-Jones 1995a; Luangsa-ard et al. 2008), stromata of *O.brunneinigra* (Hemipteran host) were flexuous, arising from between the head and the thorax of the host (Luangsa-ard et al. 2018), stromata of *O.spataforae* Tasanathai, Thanakipipattana, Khonsanit & Luangsa-ard were cylindrical, cream to pale brown (Luangsa-ard et al. 2018). However, from previously reported Hemipteran hosts, only a few hosts of the *O.sobolifera* clade were cicada nymphs in *Ophiocordyceps* (Kobayasi and Shimizu 1963; Sung et al. 2011; Crous et al. 2019). In this study, the host of *O.hydrangea* was a cicada nymph. More interestingly, the *O.hydrangea* was significantly more beautiful than other species; the stroma grew from the head of the host cicada nymph, and the top of the stroma like a hydrangea (Sung et al 2007, 2011; Crous et al. 2019). Coleoptera hosts were common in species of *Ophiocordyceps*. More than 20 species of *Ophiocordyceps* were parasitic on Coleoptera larvae (Shrestha et al. 2016). These species included *O.acicularis* (Ravenel) Petch (Petch 1933), *O.annulata* (Kobayasi & Shimizu) Spatafora, Kepler & C.A. Quandt (Kobayasi and Shimizu 1982; Spatafora et al. 2015), *O.aphodii* (Mathieson) G.H. Sung, J.M. Sung, Hywel-Jones & Spatafora (Mathieson 1949; Sung et al. 2007), *O.brunneipunctata* (Hywel-Jones) G.H. Sung, J.M. Sung, Hywel-Jones & Spatafora (Hywel-Jones 1995b; Sung et al. 2007; Luangsa-ard et al. 2008), *O.furcatosubulata* H. Yu, Y. Wang & Y.B. Wang (Wang et al. 2021a), *O.houaynhangensis* Keochanpheng, Thanakitp., Mongkols. & Luangsa-ard (Crous et al. 2018), *O.langbianensis* T.D. Lao, T.A.H. Le & N.B. Truong (Lao et al. 2021), *O.melolonthae* (Tulasne & C. Tulasne) G.H. Sung, J.M. Sung, Hywel-Jones & Spatafora (Sung et al. 2007), and *O.ravenelii* (Berkeley & M.A. Curtis) G.H. Sung, J.M. Sung, Hywel-Jones & Spatafora (Sung et al. 2007). Most species with Coleopteran host occur in soil and have solid, cylindrical, and yellow stromata. This is consistent with the results of this study.

Phylogenetic analyses based on the data from five genes showed that our phylogenetic framework of *Ophiocordyceps* was consistent with previous studies (Sung et al. 2007, 2011; Quandt et al. 2014; Simmons et al. 2015; Crous et al. 2018, 2019; Wang et al. 2018, 2021a; Lao et al. 2021). The genus of *Ophiocordyceps* consists of four clades, including the *Hirsutella* clade, *O.sobolifera* clade, *O.sphecocephala* clade, and *O.ravenelii* clade. Phylogenetic analyses showed that *O.hydrangea* clustered with *O.sobolifera*, *O.longissima*, and *O.yakusimensis* in the *O.sobolifera* clade, and *O.bidoupensis* clustered with *O.houaynhangensis*, *O.brunneipunctata*, *O.langbianensis*, *O.cossidarum*, and *O.furcatosubulata* in the same clade. Species within the *O.sobolifera* clade had different hosts, and morphological characteristics. These two new species clustered in two separate subclades within the *O.sobolifera* clade. The hosts of one subclade were cicada nymphs with stromata cylindrical or sarciniform, bright-colored, conidia were macro (Kobayasi and Shimizu 1963; Crous et al. 2019), and the hosts of another subclade were Coleoptera, with stromata cylindrical, conidia small, and a sterile apex on top of the stroma (Hywel-Jones 1995b; Luangsa-ard et al. 2008; Crous et al. 2018; Lao et al. 2021; Wang et al. 2021a). Therefore, the species of the *O.sobolifera* clade could be divided into two separate subclades when more materials were collected.

The species of *O.sobolifera* clade had diverse morphological characteristics (Table 2). The entomopathogenic fungi with cicada nymph hosts shared similar characteristics, stromata solitary or multiple, cylindrical, and bright-colored. However, they also differed in morphology. For example, *O.sobolifera* lacked a protruding ostiole with immersed perithecia (Kobayasi and Shimizu 1963), and this seems to be contrary to *O.yakusimensis* (Kobayasi and Shimizu 1963). Stromata of *O.longissima* were longer than other species, and had a short neck in perithecia (Sung et al. 2011). Compared to the ovoid perithecia of *O.longissima* and *O.yakusimensis*, *O.khonkaenensis* was flask-shaped (Crous et al. 2019). The top of the stroma of *O.hydrangea* was similar to hydrangea, the size and shape of conidiogenous cells and conidia were different from *O.khonkaenensis* (Table 2). The entomopathogenic fungi using Coleoptera hosts shared similar characteristics, such as stromata solitary, cylindrical, sterile apices on top, bright-colored. However, they had different shape and size of perithecia, asci, ascospores, conidiogenous cells, and conidia. The perithecia of *O.bidoupensis* was pyriform to lanceolate and brown-yellow. It was similar to *O.brunneipunctata*, *O.furcatosubulata*, and *O.langbianensis*, and only *O.houaynhangensis* was clavate (Hywel-Jones 1995b; Luangsa-ard et al. 2008; Crous et al. 2018; Lao et al. 2021; Wang et al. 2021a). Conidiogenous cells of *O.bidoupensis* were cone-shaped, forming on hyphae, with a hypertrophic base, tapering abruptly into a thin neck, smooth-walled, with a smaller thin neck (0.42 µm wide) than *O.brunneipunctata* (0.5 µm), *O.furcatosubulata* (0.9 µm), and *O.houaynhangensis* (0.5 µm).

Due to the unique geographical locations and climate conditions in China and Vietnam, these areas contain a rich species diversity of *Cordyceps* s.l. However, our survey of *Cordyceps* s.l. in China and Vietnam only represented a small portion of the total. More samples of *Cordyceps* s.l. will continue to be collected in China and Southeast Asia in order to uncover additional undescribed taxa, and revise species with the incorrect classification position of this group.

## Supplementary Material

XML Treatment for Ophiocordyceps hydrangea

XML Treatment for Ophiocordyceps bidoupensis
